# Retropharyngeal abscess in a post-lung transplant cystic fibrosis patient with prior cervical fusion: a case study

**DOI:** 10.1186/s12890-020-01269-6

**Published:** 2020-08-24

**Authors:** Sigrid Ladores, Leigh Ann Bray, Janet Brown, Jessica Corcoran, Jeremy Jordan, Erin Buczek

**Affiliations:** 1 University of Alabama at Birmingham School of Nursing, 1720 2nd Avenue South, NB470L, Birmingham, AL 35294-1210 USA; 2 University of Alabama at Birmingham School of Medicine, 1720 2nd Avenue South, FOT1155, Birmingham, AL 35294-3412 USA

**Keywords:** Cystic fibrosis, Post lung-transplant complications, Retropharyngeal abscess, Case report, Dental surgery

## Abstract

**Background:**

Cystic fibrosis (CF) is a chronic, genetic, incurable disease that affects primarily the respiratory and gastrointestinal systems. End-stage lung disease is the leading cause of death in people with CF, and lung transplant is required to preserve life. Anti-rejection medications are necessary post-transplant; however, these medications lower immune response and increase susceptibility to bacterial infections. Complications from infections post lung-transplant account for approximately 30% of CF-related deaths. Retropharyngeal abscess (RPA) is a rare deep neck infection that occurs most commonly in children. This is the case of a 45-year-old Caucasian male with CF who developed a retropharyngeal abscess post wisdom teeth extraction that seeded into hardware from a previous cervical disc fusion.

**Case presentation:**

The patient presented to the emergency department with severe neck and shoulder pain, limited range of motion in his arm and neck, and dysphonia. He reported feeling pain for 10 days and suspected the pain was caused by a weightlifting injury. The patient reported low-grade fever 5 days prior, which responded to acetaminophen. He was afebrile upon admission and in no respiratory distress. Diagnostic labs revealed WBC 22,000/uL and CRP 211 mg/L. The CT scan showed a large abscess in the retropharyngeal space between C2-C7. The immediate concern was airway obstruction and need for possible intubation or tracheostomy. The patient was transferred to ENT service with neurosurgery and transplant consults. The RPA was drained and lavaged. The cervical hardware was discovered to be infected and was removed. The source of the RPA infection was determined to be from the patient’s wisdom teeth extraction 6 months prior to RPA. The patient received 8 weeks of intravenous ceftriaxone for *Streptococcus pneumoniae* bacteremia and underwent revision of his cervical fusion 3 months after hardware removal.

**Conclusions:**

Clinicians should consider prophylactic antimicrobial therapy for immunocompromised patients when they are at increased risk for transient bacteremia such as following invasive procedures (e.g., tooth extraction). Prophylactic antimicrobial therapy could prevent potentially life-threatening infections such as RPA in immunocompromised patients.

## Background

Cystic fibrosis (CF) is a chronic, genetic, incurable disease characterized by a mutation in the cystic fibrosis transmembrane conductance regulator gene that results in abnormal ion transport [1]. The abnormal ion transport is responsible for the thickened mucus that results in the multi-organ system disease seen in patients with CF [1]. The median predicted life expectancy for people with CF has steadily increased from 31 years in 2002 to 47 years in 2016 [1]. Even with the increase in life expectancy, end-stage lung disease is the leading cause of death in people with CF, and transplant is required to preserve life [2]. Lung transplant survival rates are 70% at 5 years and 50% at 10 years [2]. After lung transplantation, anti-rejection medications can impede mucociliary clearance by disrupting the balance of cilia, periciliary fluid, and mucus leading to inflammation and infection [3]. Infectious complications account for about 30% of deaths post lung-transplant [4]. A retropharyngeal abscess (RPA) is a rare, life-threatening infection seen most commonly in children [5]. RPA is one of the most life-threatening deep neck infections due to the increased risk of airway obstruction, aspiration pneumonia, jugular venous thrombosis, and sepsis [5]. Morbidity and mortality rates of deep neck infections are approximately 2.6% [5]. In children, a RPA typically occurs after a respiratory infection, but in adults, the most common causes of a RPA are penetrating trauma (e.g., foreign body ingestion or oral surgery) and odontogenic sepsis [5, 6]. In adults, risk factors for RPA include chronic steroid use, excessive antibiotic use, and immunosuppressive conditions [6]. Cases of RPA caused by ingestion of fish and chicken bones [5], and wisdom teeth removal [6] have been reported in healthy adults; however, RPA has not be reported in an immunosuppressed patient with CF with seeding into the cervical hardware from previous spinal fusion. This is a case report of a middle-aged man with CF post-lung transplant and post-cervical fusion who developed a RPA post-wisdom teeth removal. Institutional Review Board approval was not sought because this is a case report on one individual who provided written informed consent.

## Case presentation

A 45-year-old Caucasian male with CF presented to the emergency department with severe neck and shoulder pain, limited range of motion in his neck and arm, and dysphonia. The patient reported feeling pain for the past 10 days and suspected the pain was due to a muscle injury from lifting weights. The patient’s primary concern was unresolved pain and difficulty speaking. He reported a low-grade fever 5 days prior, which responded to acetaminophen. He reported severe pain, which was not effectively controlled with ibuprofen or acetaminophen. Current medication regimen included Myfortic, tacrolimus, prednisone, ganciclovir, sulfamethoxazole/trimethoprim, and pancreatic enzymes.

### Past medical history

The patient was suspected to have CF shortly after birth due to a meconium ileus. Genetic testing revealed the following CF mutations: F508del and c.3846G > A. He had several distal intestinal obstructions during infancy and childhood that required surgery. Recent past medical history included bilateral lung transplant 6 years ago, cervical fusion 4 years ago to treat paresthesia of fingertips caused by spinal cord compression, and wisdom teeth extraction 6 months ago.

**Fig. 1:**
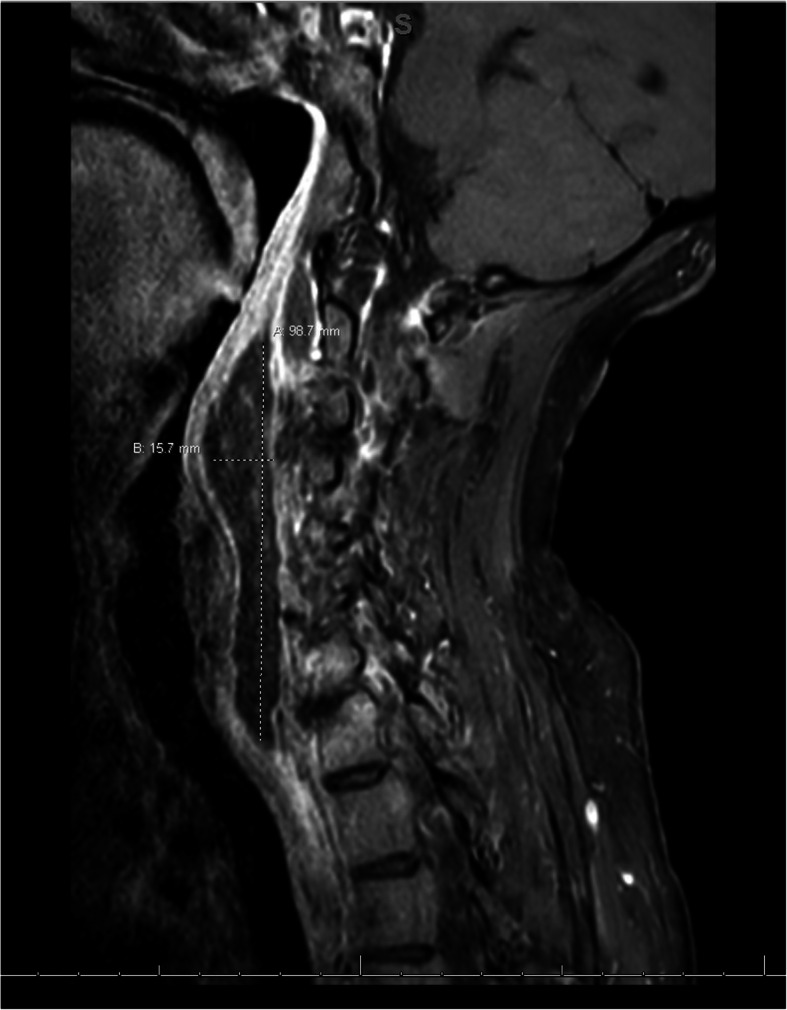
Sagittal MRI T1 fat suppression protocol with contrast, performed on admission (pre-operatively)

**Fig. 2:**
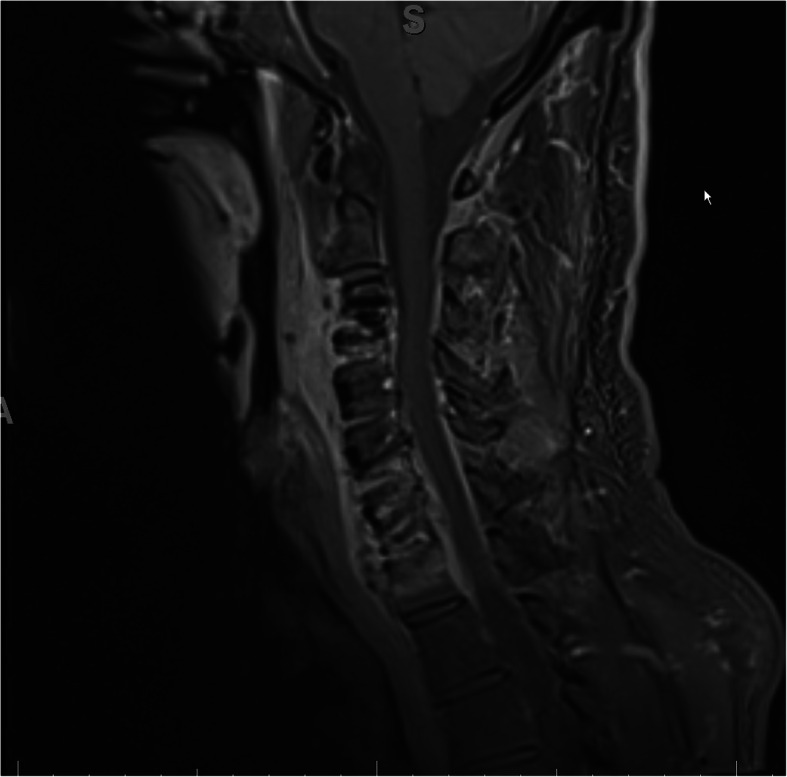
Sagittal MRI T1 fat suppression protocol with contrast performed 1 month after surgical drainage and antibiotic therapy

The patient was afebrile upon admission. Physical examination revealed enlarged cervical lymph nodes, no apparent throat mass, and no respiratory distress. Diagnostic labs revealed WBC 22,000/uL, CRP 211 mg/L, and INR 6. Initial differential diagnosis included bacterial or viral throat or upper respiratory infection. Cross sectional imaging revealed a large abscess in the retropharyngeal space between vertebrae C2 and C7 with meningeal inflammation and spinal cord compression (Fig. 1). The immediate concern was airway obstruction and need for possible intubation. The patient was admitted under the Otolaryngology (ENT) service with consults to neurosurgery and transplant teams. The neurosurgery team suggested immediate surgery due to spinal cord compression, but the markedly elevated INR which suggested significant sepsis and difficulty clotting prevented the team from moving forward with surgery. The patient was started on Zosyn as broad-spectrum antibiotic coverage until the infecting organism could be isolated. Blood cultures were drawn and revealed *Streptococcus pneumoniae* bacteremia. The patient was then switched to high-dose ceftriaxone. The immunosuppressive regimen included Myfortic, tacrolimus, and prednisone. The tacrolimus dosage was slightly reduced to keep the patient on the lower side of therapeutic immunosuppression (five with a range from five to eight). After 48 h of IV antibiotics, the RPA was drained, cultured, and irrigated by the ENT team using a transcervical approach. Around 50mls of purulent fluid was drained from the prevertebral/retropharyngeal space. The hardware from prior cervical fusion was discovered to be infected. The hardware was loose due to infection within the bone itself. The anterior cervical internal fixation hardware from vertebrae C3 through C7 was removed by the neurosurgical spine team as it was a source of infection and had become unstable. The patient received 8 weeks of high dose intravenous ceftriaxone for *S. pneumoniae* bacteremia. The source of infection was determined to be from the patient’s wisdom teeth extraction 6 months prior to RPA. The patient followed up with the ENT team to assess abscess healing 1 week after discharge and followed up with infectious disease and transplant teams 4 weeks post discharge. Subsequent MRI 1 month later showed resolution of the abscess and acute inflammation (Fig. 2). The patient’s immunocompromised status complicated the healing of the RPA. The length of intravenous antibiotics had to be extended two additional weeks to fully treat the abscess. The patient’s prior cervical fusion also complicated the healing process as the hardware had to be re-installed 3 months after removal due to new onset of paresthesia of fingertips secondary to nerve compression.

### Patient perspective

The experience of having a RPA was scary. Initially, I thought I just had a muscle injury. To go from thinking I had a simple injury to needing surgery was a shock. When the doctors realized I had a RPA, they were very adamant about the need for intubation, but I did not want to be intubated unless it was absolutely necessary. I refused elective intubation and luckily everything turned out fine. I had to stay on IV antibiotics at home for longer than expected due to my transplant medications. Overall, the experience was scary, and I am glad that chapter is behind me.

## Discussion and conclusions

Patients with CF, especially post-lung transplant, are at an increased risk for upper airway disorders such as chronic sinusitis and nasal polyps due to the inherent mucociliary clearance disturbance caused by CF pathophysiology and long-term use of anti-rejection medications [3]. This increased risk of upper airway disorders may predispose healthcare providers to misdiagnose a patient with CF with more common disorders such as sinusitis or nasal polyps, which may make RPA more difficult to diagnose [5]. The relative infrequency of RPA may also predispose healthcare providers to exclude RPA in the differential diagnosis for patients with CF presenting with upper airway symptoms. Additionally, patients with CF are accustomed to frequent sinusitis and nasal polyps decreasing the likelihood that they will report infection concerns to healthcare providers in a timely manner. The combination of anti-rejection medication from bilateral lung transplantation and hardware from prior cervical fusion compound the risk of developing an infection such as RPA in this patient. Prompt diagnosis of RPA is critical due to the potential for airway compression and respiratory arrest caused by the space-occupying abscess. Traditionally, CT scan with contrast is the optimal imaging modality for an isolated RPA. MRI was used in addition to CT for this patient due to history of spine surgery and neurologic sequelae on presentation. Prompt Otolaryngology evaluation with awake flexible fiberoptic laryngoscopy is also important to assess airway patency. A RPA, if not treated early and appropriately, can lead to bacteremia and even death [5]. Post-transplant patients with CF are at an increased risk for overwhelming bacterial infections due to their chronic immunocompromised status [5]. Therefore, RPA has an increased rate of morbidity and mortality in immunocompromised patient populations, such as the post-transplant CF population [5]. Aggressive airway management to prevent airway compromise and antimicrobial therapy to prevent infection is necessary to decrease morbidity and mortality in this population. Prescribing an effective antibiotic regimen in patients with CF can be difficult due to the frequent long-term administration of antibiotics required post lung transplantation leading to colonization with multi-drug resistant bacteria (i.e., *Pseudomonas aeruginosa)* in some patients [7]. The RPA presented 6 months post wisdom teeth extraction. A similar case study reported a deep neck infection post dental surgery that also presented 6 months post dental procedure [8].

### Strengths and limitations

Case studies offer insight into a specific phenomenon that has not yet been well documented [9]. The in-depth understanding of this patient’s experience with a RPA post-lung transplant is a strength of this case study. A limitation of this case study is the lack of generalizability of the experience to other patients from other populations.

In conclusion, current guidelines state that clinicians should consider prophylactic antimicrobial therapy for immunocompromised patients in situations where these patients are at increased risk for transient bacteremia such as following invasive procedures (e.g., tooth extraction). Prophylactic antimicrobial therapy could prevent potentially life-threatening infections such as RPA in immunocompromised patients. The dentist plays a vital role in consulting with the immunocompromised person’s healthcare team to determine acceptable antibiotic and dosage for prophylaxis given the patient’s history [7]. It is important to consider the patient’s current and past medical history to identify the most appropriate prophylactic antibiotics to prescribe before invasive procedures. Healthcare providers of medically fragile patients must be cognizant of complex disease processes and their interrelation with body systems when diagnosing and treating immunocompromised patients.

## Data Availability

Data sharing is not applicable to this article as no datasets were generated or analysed during the current study.

## Abbreviations

CF: Cystic Fibrosis
RPA: Retropharyngeal Abscess
